# DOG1 as a novel antibody-drug conjugate target for the treatment of multiple gastrointestinal tumors and liver metastasis

**DOI:** 10.3389/fimmu.2023.1051506

**Published:** 2023-01-26

**Authors:** Yangping Wu, Wenting Li, Xiangzheng Chen, Haichuan Wang, Siyuan Su, Ying Xu, Xiangbing Deng, Tinghan Yang, Mingtian Wei, Li Li, Yixin Liu, Jinliang Yang, Weimin Li

**Affiliations:** ^1^ Targeted Tracer Research and Development Laboratory, Department of Respiratory and Critical Care Medicine, State Key Laboratory of Biotherapy and Cancer Center, West China Hospital, Sichuan University, Chengdu, China; ^2^ State Key Laboratory of Biotherapy and Collaborative Innovation Center for Biotherapy, West China Hospital, Sichuan University, Chengdu, China; ^3^ Department of Liver Surgery & Liver Transplantation, State Key Laboratory of Biotherapy and Cancer Center, West China Hospital, Sichuan University, Chengdu, China; ^4^ Department of Chemistry, University of Illinois Chicago, Chicago, IL, United States; ^5^ Department of Gastrointestinal Surgery, West China Hospital, Sichuan University, Chengdu, China; ^6^ Institute of Clinical Pathology, West China Hospital of Sichuan University, Chengdu, China; ^7^ Department of Thoracic Surgery, West China Hospital, Sichuan University, Chengdu, China

**Keywords:** DOG1, GIST, antibody-drug conjugate, alimentary tract cancers, liver metastasis

## Abstract

Discovered On Gastrointestinal stromal tumors protein 1 (DOG1), a major calcium-activated chloride channel, has been used as a common diagnostic marker for gastrointestinal stromal tumors. However, the therapeutic application of DOG1 was not well defined. Here, we aim to investigate its potential as a therapeutic target for an antibody-drug conjugate (ADC) in various cancers of the alimentary tract and metastasis. The DOG1 expression profile was determined among TCGA samples and tissue microarrays. High levels of DOG1 expression were ubiquitously observed in multiple cancer samples from the alimentary tract determined by TCGA samples and tissue microarrays. Circulating tumor cells isolated from metastatic colon cancer patients were also positive for DOG1 expression. The mechanisms of anti-DOG1 antibody were investigated by dual-luciferase reporter assay. The anti-DOG1 antibody could inhibit proliferation and metastasis *via* p53 signaling in limited cancer cell lines. The anti-DOG1 antibody was conjugated with a microtubule inhibitor DM4, to construct a new anti-DOG1-DM4-ADC to strengthen its activity. The anti-DOG1-DM4-ADC showed cytotoxicity at the nanomolar level *in vitro.* In the murine xenograft tumor models, treatment of anti-DOG1-DM4-ADC achieved a significant tumor growth inhibition rate. Our study indicates that anti-DOG1-DM4-ADC may be promising therapeutic molecules for DOG1-positive alimentary tract tumors and may be effective in inhibiting recurrence after curative resection of liver metastases of colorectal origin.

## Introduction

DOG1 (also known as transmembrane member 16A or anoctamin 1) is one of the major components of the calcium-activated chloride channels expressed in the plasma membranes. It is revealed in a wide variety of tissues, including the secretory epithelia (airway, intestine, and salivary glands), smooth muscle, and neurons (1–5). The DOG1 protein mediates transepithelial ion transportation, involving in the regulation of airway fluid secretion, gut motility, secretory functions of exocrine glands, renal function, vascular smooth muscle contraction, and nociception (6, 7).

DOG1 is expressed in ~99% of gastrointestinal stromal tumors (GISTs) derived from Cajal cells regardless of conventional KIT (CD117) or platelet derived growth factor receptor alpha (PDGFRA) mutation status (8). In the situation that KIT immunostaining or KIT/PDGFRA mutation analysis is defective, DOG1 is widely used as the marker for the diagnosis of GIST (9–11). In addition to GIST, increased expression of DOG1 has been reported in esophageal squamous cell carcinoma (ESCC), esophageal adenocarcinoma (EAC), diffuse gastric adenocarcinoma (DGAC), pancreatic adenocarcinoma (PAC), colorectal cancers (CRC) and head and neck squamous cell carcinomas (HNSCC) in recent years (12–14). Moreover, some studies have demonstrated the increased expression of DOG1 in hepatic metastasis from CRC (15), and others revealed that DOG1 overexpression in HNSCC could be predictive of the presence of distant metastasis (16). In this study, we provided an expanded expression profile of DOG1 in alimentary tract primary cancers and metastasis. The results revealed that DOG1 was elevated in most gastrointestinal tumors. Interestingly, the abnormal high expression of DOG1 in colon cancer liver metastasis. However, whether DOG1 can be a potential therapeutic target in the treatment of cancers of the alimentary tract and metastasis remains to be investigated.

The antibody-drug conjugates (ADCs) are composed of monoclonal antibodies conjugated with cytotoxic agents through chemical linkers. Antibodies can specifically bind to tumor cell surface antigens to form antigen-ADC immune complexes and to promote protein-mediated cell endocytosis in tumor cells (17, 18). This approach allows direct delivery of highly potent cytotoxic agents to antigen-positive tumor tissue with limited off-target toxicities (19). Since the first ADC, Mylotarg^®^ (gemtuzumab ozogamicin), was approved in 2000 by the US Food and Drug Administration (FDA) against CD33-positive acute myeloid leukemia (AML), there have been 14 ADCs received market approval (20, 21), and over 100 ADCs are currently under clinical development. However, half of these 14 ADCs approved by FDA are mainly used against hematological malignancies such as AML and anaplastic large cell lymphoma (ALCL). The rest are mainly against solid tumors including Her2-positive breast cancer, Triple-negative breast cancer (TNBC), urothelial cancer and HNSCC (20–26). However, ADCs for gastrointestinal tumors/metastasis are still insufficient, thus there is a great need to identify novel targets to expand the application of ADCs for the treatment of primary tumors and metastasis.

In this study, we provided substantial evidence that DOG1 is broadly and highly expressed in various types of alimentary tract cancers/liver metastasis. We also uncovered the mechanism by which anti-DOG1 antibodies inhibit tumor growth and metastasis. Furthermore, we constructed a novel anti-DOG1 ADCs conjugated with the highly potent maytansine-based payload DM4 conjugated through the cleavable linker SPDB to test its potential as a novel antitumor treatment. In addition, we detected DOG1 expression in circulating tumor cells (CTC) and established an experimental liver metastasis model of colon cancer to evaluate the potential of the anti-DOG1 ADCs in the prevention and treatment of liver metastasis. The results suggested that DOG1 may be a potential therapeutic target and DOG1-ADCs may be a promising novel targeted drug against alimentary tract cancers/liver metastasis.

## Methods

### Human samples and tissue microarrays

Clinical surgically resected specimens from patients with liver cancer, colon cancer and GIST, as well as samples from patients with colon cancer were obtained from the biological sample bank of West China Hospital, Sichuan University. All human samples were isolated following approved clinical protocols and in accordance with the Ethics Board of West China Hospital, Sichuan University approval and informed consent from patients. TMAs were purchased from US Biomax and Shanghai Outdo Biotech (mainly from Asian donors).

### Cell lines

Human liver cancer HepG2, HCC-LM3, and SNU-449 cell lines; colon cancer HT-29, HCT-116, LoVo, and SW-620 cell lines; gastric cancer NCI-N87, MGC-803, MKN-45, and AGS cell lines; and esophageal cancer Eca-109 cell lines were obtained from the American Type Culture Collection and the Chinese National Infrastructure of Cell Line Resource. Human liver cancer HCC-LM9, esophageal cancer Kyse-410, Kyse-510, and Kyse-180 and GIST882 cell lines were obtained from the Department of Gastrointestinal Surgery, West China Hospital, Sichuan University. Imatinib (IM)-resistant GIST882 cells were incubated with 10 μM IM for 3 months and maintained to confluence. HT29-luciferase-expressing cells were purchased from ZQXZBIO. The cell lines were cultured in high-glucose Dulbecco’s modified Eagle’s medium (C11995500BT, Gibco) or RPMI 1640 (C11875500BT, Gibco) supplemented with 10% fetal bovine serum (10099141C, Gibco) at 37°C in 5% CO_2_. The FreeStyle 293-F cell line (R79007, Gibco) was expanded in FreeStyle 293 Expression Medium (12338018, Gibco). Mycoplasma testing was performed annually using the Mycoalert Testing Kit (NC9719283, Thermo Fisher Scientific).

### Flow cytometry

The tumor cell lines mentioned above cultured in proper medium were trypsinized, washed and incubated with anti-DOG1 antibodies or human IgG isotype control (02-7102, Invitrogen) at a concentration of 10 μg/ml at 37°C for 45 min. The samples were washed and incubated with FITC-conjugated AffiniPure Goat Anti-Human IgG (H+L) (SA00003-12, Proteintech) at 1:50. After incubation, the cells were washed and resuspended in 500 μl of PBS. The fluorescence intensity of FITC was determined by flow cytometry (Novocyte 2060R, ACEA).

### CTC detection in blood samples from colon cancer patients

Peripheral blood samples (4 ml) were collected in heparinized tubes (367871, BD Biosciences) after discarding the first 2.5 ml of blood (27–30). Samples were stored at 0–4 °C until further processing within 24 h after blood sampling. Erythrocytes were removed using red blood cell lysis buffer (154 mM NH_4_Cl, 10 mM KHCO_3_, and 0.1 mM EDTA). The remaining cells were resuspended in 1 ml of staining buffer (0.5% bovine serum albumin (BSA), 2 mM EDTA in PBS), split into two equal fractions, and stained with specific antibodies against EpCam (APC-labeled, 324207-25, Biolegend), CD45 (Alexa Fluor 488-labeled, 53-9458-82, eBioscience) and DOG1 (Alexa Fluor 405-labeled, clone SPM580, NBP2-34812AF405, Novus Bio) or the relevant isotype control antibodies (BD Biosciences). The cells were fixed with 80% methanol (5 min) and then permeabilized with 0.1% PBS-Tween for 20 min. During the permeabilization step, the cells were stained with anti-pan cytokeratin antibody (eFluor 570-labeled, 41-9003-80, eBioscience) or the relevant isotype control (BD Biosciences). After staining, the cells were washed with PBS and immediately tested on a CytoFLEX Flow Cytometer (Beckman Coulter). Data were analyzed with Kaluza Analysis 2.1.

### Preparation and characterization of ADCs

The anti-DOG1 antibodies and DM4-SPDB were mixed in a molar ratio of 1:10 in conjugation buffer (50 mM potassium phosphate, 50 mM sodium chloride, 2 mM EDTA, pH 7.2) and stirred at 25°C overnight^20^. After centrifugation at 4000 g for 10 min in an ultrafiltration concentrator (VS15T21, Sartorius), the unconjugated DM4-SPDB was removed, and the anti-DOG1 antibody-DM4 conjugates were replaced with storage buffer (50 mM sodium phosphate, 50 mM sodium chloride, pH 7.2). The drug-antibody ratio (DAR) of anti-DOG1 antibody-DM4 was established by LC-MS (Quattro Premier XE, Waters).

### Internalization analysis

Internalization of the DOG1-antibody complex was detected by flow cytometry. Various tumor cell lines were harvested with 0.25% trypsin-EDTA (25200056, Gibco) and washed with PBS. Experimental groups were incubated with 5 μg/ml anti-DOG1 antibody on ice for 1 h. Cells were washed and incubated at 37°C for 0, 1, 4 and 8 h. The control of each group was incubated with human IgG isotype control (02-7102, Invitrogen) on ice for 1 h and then washed. The cells above were all labeled with FITC-conjugated goat anti-human IgG (H+L) (SA00003-12, Proteintech) at 4°C for 1 h. Then, cells from both groups were washed and resuspended in 500 μl of PBS for flow cytometric analysis. The mean fluorescence intensity (MFI) was corrected by the control, and the internalization of the DOG1-antibody complex was calculated as the percent MFI relative to that incubated with antibody at 4°C for 1 h.

For visualization of the internalization of anti-DOG1 DM4 ADCs, DOG1-positive cells were seeded into glass bottom cell culture dishes (801002, NEST) at 1 × 10^5^ cells per well and cultured at 37°C and 5% CO_2_. After 24 h of culture, each cell type was incubated with anti-DOG1 DM4 ADC labeled with Cy5.5 at a concentration of 20 μg/ml at 4 °C for 1 h and 37 °C for 1 h, 3 h, and 6 h. Then, the cells were washed with PBS and stained for 10 min at room temperature with FITC-labeled phalloidin (P5282, Sigma-Aldrich). After the samples were washed with PBS, the cell nuclei were labeled with Hoechst 33342 (B8040-25 mg, Solarbio). Visualization of immunofluorescence was observed with a confocal laser scanning microscope (Leica, DM-8).

### Immunoblotting

Lysates were quantified by a BCA Protein Assay Kit (ab102536, Abcam). Fifteen micrograms of protein were loaded on an 8% SDS-PAGE gel with Tris-glycine-SDS running buffer and transferred to a polyvinylidene difluoride (PVDF) membrane (88520, Thermo Scientific) by a Trans-Blot Turbo Transfer System (Bio-Rad). Primary antibodies were bought from Cell Signaling Technology, Santa Cruz and ABclonal, respectively. The anti-mouse (7076S) and anti-rabbit (7074S) HRP-conjugated secondary antibody were purchased from Cell Signaling Technology. The protein bands were scanned and analyzed using ChemDoc MP system (Bio-Rad).

### *In vitro* cytotoxicity assay

Cytotoxicity was determined by real-time cell analysis (RTCA) analyzers (xCELLigence RTCA SP, Agilent). Fifty microliters of culture medium were added to each well of an E-plate 96 for background adjustment. Cells were added at a density of 10,000 to 20,000 cells per well (changes according to the growth rate of different cells) and incubated until the cell index (CI) was above 0.8. Then, the test drug was diluted in the culture medium to different concentrations. Untreated cells were set as the control. Triplicate samples were measured for each concentration. Data were analyzed with GraphPad Prism 8.0 software using a four-parameter logistic nonlinear regression model.

### GIST PDX models

Animal experiments were approved by the Committee of Animal Care of the West China Hospital Sichuan University (Ethical approval number, 2020368A). For the GIST PDX model, 6- to 10-week-old NOG mice (HFK Biotechnology) were subcutaneously implanted with 2- to 3-mm fragments of surgical specimens from GIST patients into each hind side of the flank^21^. All experiments described herein were performed using PDXs from passages 1 to 4. When the average tumor volumes reached approximately 200 mm^3^, the mice were randomized into groups (N = 5 to 8 mice per group). Then, 10 mg/kg anti-DOG1 antibodies, 5 mg/kg anti-DOG1 DM4 ADC, 10 mg/kg anti-DOG1 DM4 ADC and control (PBS) were administered *via* tail vein injection to the mice once every three days for a total of three doses.

### CDX models for multiple human cancer types

Six-week-old female BALB/c nude mice were given a single subcutaneous inoculation of HepG2, HT-29, MGC-803 and Kyse-410 cell suspensions (1 to 10×10^6^ cells in 100 μl of cell culture medium without serum and antibiotics) into the right flank. When the average tumor volume reached approximately 100–200 mm^3^, the mice were randomized into groups (N = 5 to 8 mice per group). Then, 10 mg/kg anti-DOG1 antibodies, 5 mg/kg anti-DOG1 DM4 ADC, 10 mg/kg anti-DOG1 DM4 ADC and control (PBS) were administered *via* tail vein injection to mice once every three days for a total of three doses.

### *In vivo* efficacy study in the mouse xenograft model

Tumor volume and body weight were monitored at least weekly. Tumors were measured with digital calipers in two dimensions, long and short axis (in millimeters), and tumor volume (mm^3^) was calculated using the following formula: 0.52 × long axis × short axis^2^. Data collection was stopped, and the mice were euthanized if they exhibited ≥20% weight loss, inactivity, or poor body condition; when the individual tumor volume reached ≥1000 mm^3^ for PDX models or ≥2500 mm^3^ for CDX models; or when the study reached 90 days after randomization. Efficacy was measured by calculating the %Tumor growth inhibition (TGI): %TGI = [1 - (mean tumor volume of treatment group/mean tumor volume of control group)] × 100% was determined at the time point when difference between the treatment and control groups was maximal. Values for the rate of complete response (CR) are given as the percentage of mice in a group with a tumor burden ≤ 25 mm^3^ for at least three consecutive measurements. Partial response^2^ is given as the percentage of mice in a group with a tumor burden less than half of their starting tumor volume at the time of randomization but > 25 mm^3^ for three consecutive measurements.

### Animal model for experimental colorectal cancer liver metastasis

Seven-week-old female BALB/c nude mice (HFK Biotechnology) were anesthetized by a continuous flow of 2%–3% isoflurane. For generation of mouse models with liver metastases derived from human colorectal cancer cells, HT29-luciferase-expressing cells (1 × 10^6^) were suspended in 50 μl of PBS and injected into the spleens of mice. After a one-week recovery, the mice were randomized into vehicle or treatment groups. The mice were then given endotoxin-free luciferase substrate and photographed with an IVIS Spectrum *in vivo* imaging system (Perkin Elmer) once a week.

### Signal pathway assays

Analysis of various key signaling pathways implicated in human tumorigenesis was performed using the Cignal Finder Cancer 10-pathway Reporter Array kit (CCA-001L, Qiagen). Cellular transfection was performed with Lipofectamine 3000 (L3000001, Invitrogen). The transfected cells were then incubated with vehicle or 100 nM or 200 nM anti-DOG1 antibody for another 24 h. A dual-luciferase reporter assay system (E1910, Promega) was used to obtain firefly luminescence and Renilla luminescence readings using a CLARIOstar Plus microplate reader (BMG Labtech). Firefly constructs visualized the modulation of key transcription factors, usually a downstream target of a particular signaling pathway. The Renilla construct functioned as an internal control to normalize transfection efficiencies and to monitor cell viability. Luminescence for each sample was calculated based on the firefly-to-Renilla luminescence ratio.

### Statistical analysis

Statistical analyses were performed using GraphPad Prism software. IHC data were analyzed by the Mann-Whitney test. Prognostic factors were analyzed using a univariate model and multivariate regression model. Kaplan-Meier survival statistics were calculated using the log-rank test. Between-group comparisons were analyzed by one-way ANOVA or two-way ANOVA. Differences with P < 0.05 were considered statistically significant. *P < 0.05; **P <0.01; ***P <0.001; ****P <0.0001.

## Results

### Increased expression of DOG1 among various neoplasms of alimentary tract and metastasis

**Figure 1:**
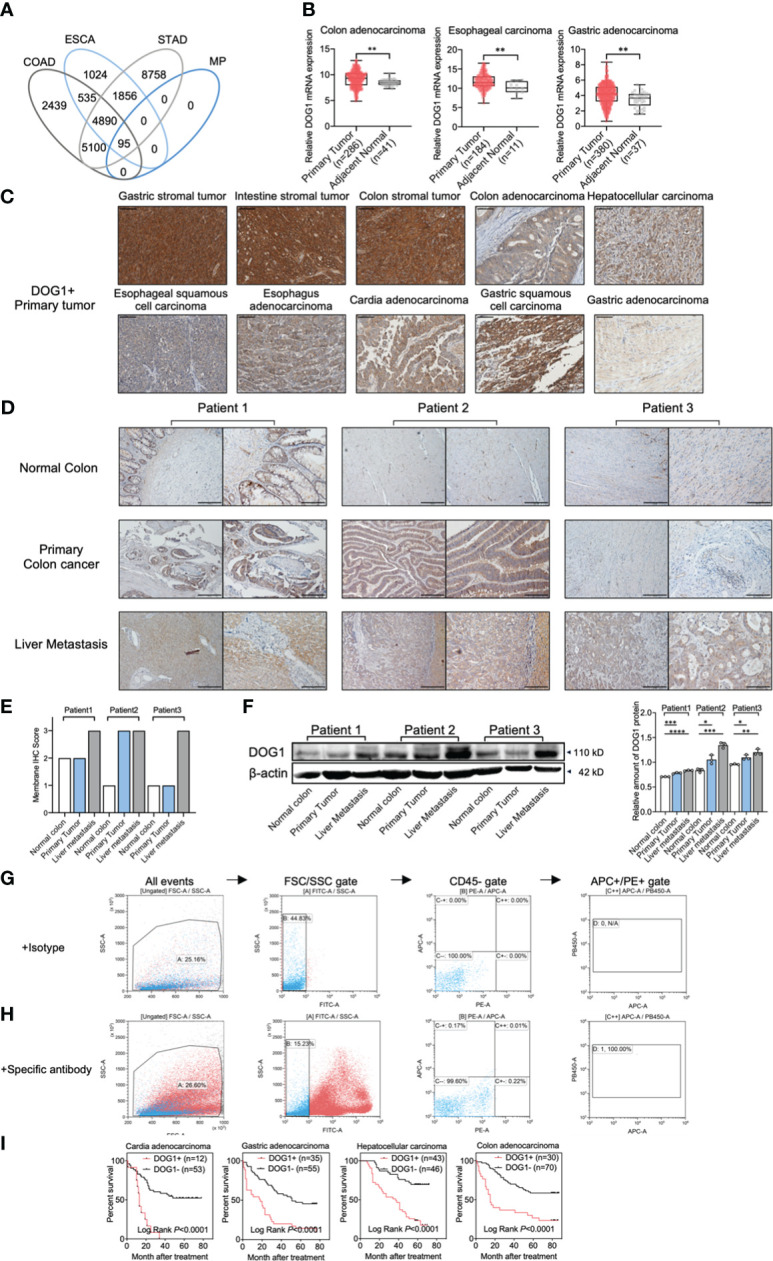
DOG1 expression analysis at the gene and protein levels in tumor tissues and CTCs. **(A)** Venn diagram showing the overlaps between the overexpressed target sets for COAD: Colon adenocarcinoma, ESCA: Esophageal carcinoma, STAD: Stomach adenocarcinoma and MP: Membrane protein; **(B)** RNA-seq data of multiple gastrointestinal cancers from TCGA analyzed by UCSC Xena (the University of California, Santa Cruz) showed DOG1 RNA expression in primary tumors compared to normal tissues adjacent to the tumor. Axis units are log2 (normalized count+1). Student’s t test; **(C)** Representative immunohistochemical images for DOG1 protein expression in primary tumors. Positive IHC staining for DOG1 is indicated by a brown precipitate. Scale bar, 50 µm; **(D)** Representative IHC for DOG1in tissue samples from colon cancer patients with liver metastasis. Scar bar is 200 µm (left) and 100 µm (right); **(E)** DOG1 IHC scores of tissue samples from colon cancer patients with liver metastasis in **(D–F)** Immunoblot of DOG1 expression in tissue samples from colon cancer patients with liver metastasis by western blot. Quantitative analysis of DOG1 protein expression in tissue samples from colon cancer patients with liver metastasis by western blot (n=3). Commercial anti-DOG1 antibody sp31 used in **(A-F) (G)** Detection of DOG1+ CTCs from colon cancer patient by flow cytometry. Erythrocytes were lysed and cells were stained with an antibody cocktail against CD45, EpCam, Pan Cytokeratin (CK10, 14, 15, 16 and 19) and DOG1 (commercial antibody NBP2-34812AF405). EpCam^+^ CK^+^ CD45^−^ tumor cells were detected by flow cytometry by first gating out the cell debris and cell clumps in the forward/side scatter plot (gate A). Then, the CD45^+^ (FITC) cells were excluded by gating at the CD45^−^ cell population (gate B). The thresholds for specific EpCam (APC) and cytokeratin (PE) signals were determined using the sample stained with the isotype control antibodies (gate C). Gates were set to have no positive events above these thresholds in the control sample (gate C++); **(H)** The same gating strategy was then applied for detecting EpCam^+^ CK^+^ CD45^−^ cells in the sample stained with the specific antibodies. DOG1^+^ (PB450) cells were sellected in gate **(D)** In this plasma sample of colon cancer patient with liver metastasis, 100% (1/1) DOG1^+^ cells were selected out in CTCs; **(I)** Kaplan–Meier survival curves for patients with cardia adenocarcinoma, gastric adenocarcinoma, hepatocellular carcinoma and colon adenocarcinoma with DOG1+/− staining are shown. Log-rank test. Comparison within groups: *P < 0.05; **P <0.01; ***P <0.001; ****P <0.0001.

First, we evaluate the importance of DOG1 in human cancers from the digestive system, including esophagus, stomach, liver and colon. The mRNA expression profiles were retrieved from The Cancer Genome Atlas (TCGA) database, and the significantly upregulated genes were identified among colon adenocarcinomas (COAD), esophageal cancers (ESCA) and stomach adenocarcinomas (STAD). A total of 95 genes encoding membrane proteins were increased among the three cohorts (**Figure 1A**). In addition, we found 33 genes whose high expression was associated with poor prognosis, including *DOG1* (Supplemental experimental materials and procedures). Consistently, increased expressions of *DOG1* were found in COAD, ESCA and STAD samples compared to adjacent normal tissues, respectively (**Figure 1B**).

Next, we determined DOG1 expression patterns with immunohistological (IHC) stainings using tissue microarrays (TMA), which included tumor specimens of common alimentary tract cancer types and the corresponding adjacent normal tissues or healthy tissues. We found a ubiquitous accumulation of DOG1 (~87% of tumor specimens) among ESCA, COAD, GIST, and gastric carcinoma samples (**Figure S1**; **Table 1**). Intriguingly, primary liver cancer (PLC) and liver metastasis samples were also positive for DOG1 staining (**Figure S2** and **Figure S3A**; **Table 1**). In contrast, normal tissues representing 42 human organs were significantly less for DOG1 staining than tumor tissues (32.9% vs. 87.3%, p< 0.0001) (**Figure S1** and **Figure S3B**).

**Table 1:** The positive expression rate of DOG1 in human tumor TMAs.

Cancer type	Total TMA numbers	Positive numbers	Positive rate
Gastrointestinal stromal tumor	30	28	93.3%
Cardia adenocarcinoma	25	23	92%
Liver metastasis	34	31	91.2%
Gastric adenocarcinoma	28	25	89.3%
Colon adenocarcinoma	105	91	86.7%
Primary hepatocellular carcinoma	75	65	86.7%
Gastric squamous cell carcinoma	14	12	85.7%
Esophageal squamous cell carcinoma	129	109	84.5%
Esophageal adenocarcinoma	14	11	78.6%
Total	454	395	87%

TMAs of different tumor types were evaluated for DOG1 expression by IHC and scored on a range of 0 to 3. An IHC score of ≥ 2 was chosen to identify tumors with positive DOG1 expression.

To substantiate these findings, we collected tumor tissue specimens from the biological sample bank of West China Hospital and performed IHC stainings. We found that DOG1 was positively expressed in GIST, colon cancers and PLC with positive rates of 97.1% (33/34), 86.1% (31/36), and 85.7% (6/7) respectively (**Figure 1C**). Surprisingly, we observed that the liver metastasis from primary colon cancer remained a high expression of DOG1 in 3/3 paired patient samples through IHC scores (**Figures 1D, E**). While semiquantitative methods exist for IHC, much is left to be desired in terms of reproducibility and agreement between laboratories and between pathologists scoring slides on a scale of 0, +, ++, +++ (31). Thus, we further confirmed this observation *via* protein quantification by Western blotting. The DOG1 expression levels in primary colon cancer tissues and liver metastatic tissues were higher than those from paired normal colon tissues (**Figure 1F**).

Circulating tumor cells (CTCs) are now considered to be a risk factor for tumor recurrence and metastasis. In this study, we detected CTCs (CD45 [-], pan-CK [+], EPCAM [+]) from plasm samples of 9 colon cancer patients. Interestingly, 66.7% (6 of 9 patients) were CTCs positive, and all CTCs separated from 6 positive patients were positive express DOG1 (**Figures 1G, H**; **Figure S3C**), potentiating the therapeutic implications of DOG1 as a broad-spectrum biomarker. Furthermore, univariate and multivariate analyses of patients with cardia adenocarcinoma, gastric adenocarcinoma, HCC, and COAD showed that high DOG1 staining was significantly predictive of poor survival (**Figure 1I**).

Altogether, the present findings suggest the ubiquitous upregulation of DOG1 among the tumors from alimentary tracts and high expression of DOG1 is associated with poor survival outcome.

### Targeting DOG1 inhibits cell migration and transition through p53

**Figure 2:**
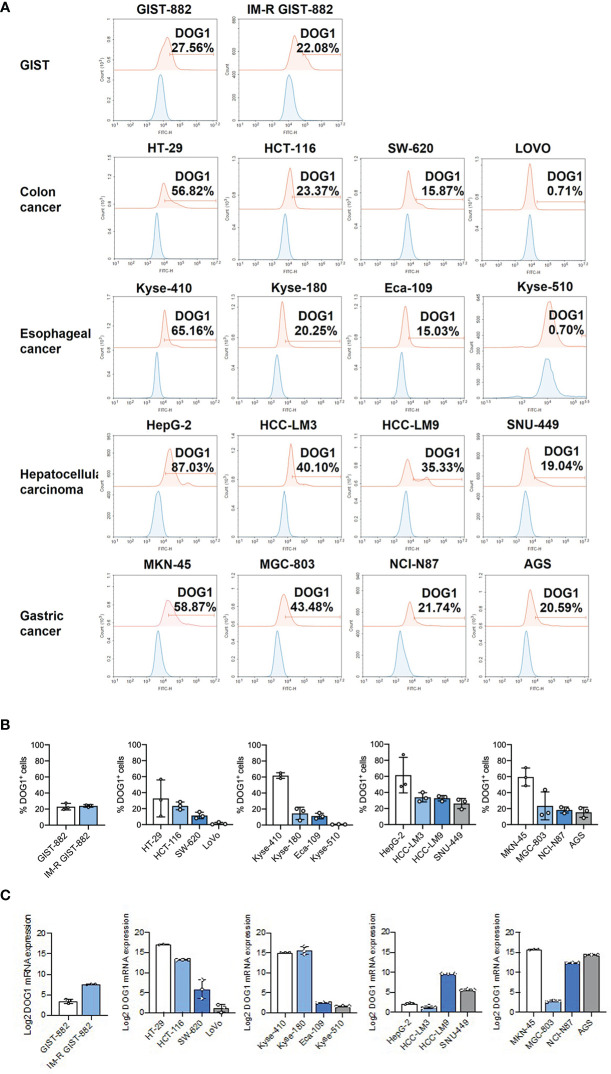
DOG1 was expressed on the cell surface. **(A)** Representative graphs of DOG1 surface expression analyzed by flow cytometry. Blue is the isotype control group, and red is the anti-DOG1 antibody group; **(B)** Flow cytometric detection of DOG1 protein expression on the surface in various tumor cell lines, including GIST cells and colon, esophageal, liver and gastric cancer cells. Three independent experiments were performed; **(C)** DOG1 mRNA expression in various tumor cell lines, including GIST cells and colon, esophageal, liver and gastric cancer cells, were detected by qPCR. Three independent experiments were performed.

**Figure 3:**
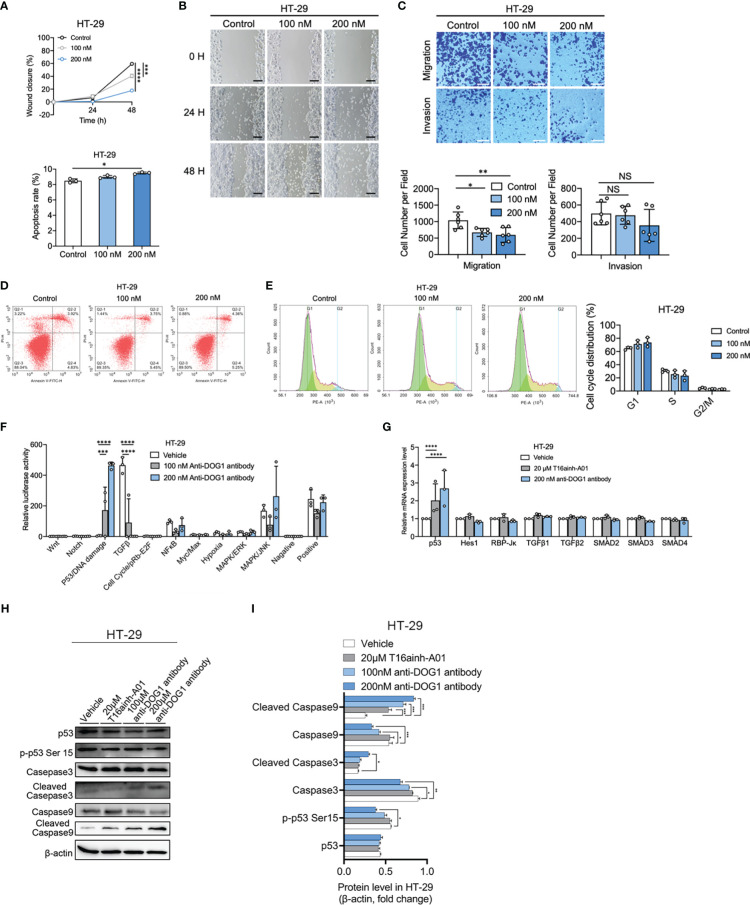
Anti-DOG1 antibody induced cell apoptosis and inhibited cell migration and invasion through p53 signaling pathway in HT-29 colon cancer cells. **(A)** The wound closure for HT-29 and GIST-882 was quantified at every 24 h post-wound (mean ± S.E.M., n = 6); **(B)** Representative image of wound healing assay in HT-29 cells at 0 h, 24 h and 48 h post wounding. The cells were treated with 100 nM and 200 nM anti-DOG1 antibody. Scale bar, 500 μm; **(C)** Migration (without Matrigel) and invasion (with Matrigel) of HT-29 cells were suppressed by the anti-DOG1 antibody compared with the control as shown by Transwell assays. Representative images are shown. Scale bar, 100 µm. Bar graphs of panel C are shown. Values are the mean ± SD; n=6; **(D)** Anti-DOG1 antibody-induced apoptosis in HT-29 cells. Apoptotic cells were quantified by Annexin V/PI double staining assay. HT-29 cells are treated with vehicle, 100 nM and 200 nM anti-DOG1 antibody for 48 h. Analysis on cell apoptosis results of I (n=3); **(E)** Flow cytometric analysis of the cell cycle distribution in HT-29 cells treated with vehicle, 100 nM and 200 nM anti-DOG1 antibody for 48 h. Bar graphs showing an increase of G1 phase and a decrease of S phase in cell cycle for the percentage of indicated cells in K (n=3), but without statistical significance; **(F)** Expression levels of 10 major cell signaling pathways in HT-29 cells treated with anti-DOG1 antibody. Values are the mean ± SD; n=3; **(G)** Relative mRNA-level of p53, Notch and TGFβ signaling of HT-29 cells after 48 h treatment with vehicle, 20 μM T16ainh-A01 and 200 nM anti-DOG1 antibody as determined by qRT-PCR. Data are normalized to the respective vehicle control and represent the mean ± SD; n=3; **(H)** Immunoblots of lysates from the HT-29 cell lines after 48 h treatment with vehicle, 20 μM T16ainh-A01, 100nM and 200 nM anti-DOG1 antibody. β-actin was used as control; **(I)** Bar graph showing quantitative analysis of protein expressions (n=3). Data were normalized by β-actin. Compared with the control group by one-way ANOVA. *P < 0.05; **P <0.01; ***P <0.001; ****P <0.0001.

First, to evaluate the potentiality of DOG1 as a therapeutic target, the anti-DOG1 antibody was first applied *in vitro*. Expression levels of DOG1 were first determined among a panel of human alimentary tract cancer (GIST, HCC, ESCA, GSAD and COAD) cell lines by flow cytometry. The results showed that anti-DOG1 antibody could bind to DOG1 protein on the cell surface of GIST882, HepG2, Kyse-410, MKN45, and HT-29 cell lines with a relatively high positive rate (**Figures 2A, B**). Consistently, the DOG1 mRNA levels detected by RT-qPCR were paralleled with the protein levels (**Figure 2C**; **Figure S3D**). Next, we treated these DOG1(+) cell lines against with different concentration of anti-DOG1 antibody (100nM and 200nM). The results demonstrated that anti-DOG1 antibody could induced cell apoptosis and inhibited proliferation as well as migration in HT-29, but no significant differences in cell invasion (**Figures 3A-C**). In GIST cell line, anti-DOG1 antibody appears to have no effect on proliferation but could affect cell invasion and migration (**Figure S4A, B**). The rest cell lines were no statistical difference after DOG1 antibody treatment (Date not shown). Moreover, anti-DOG1 antibody could arrested HT-29 cells at G1 phase of cell cycle, but without statistical significance (**Figures 3D, E**).

Next, we investigated the biochemical mechanisms of anti-DOG1 antibody in regulating biological activities of tumor cells. To this end, we performed signaling pathway screening assays in HT-29 COAD cell lines upon the administration of anti-DOG1 antibody (100nM and 200nM). Intriguingly, compared to the vehicle control treated cells, the DOG1 blocked HT-29 cells significantly activated P53/DNA damage signaling and inhibited TGFβ signaling (**Figure 3F**). Subsequent analysis by RT-PCR validated the upregulation of *P53* mRNA levels in the anti-DOG1 treated HT-29 COAD cells (**Figure 3G**). However, no evident changes of *TGFβ1*, *TGFβ2* or the downstream effectors *SMAD2*, *SMAD3* and *SMAD4* levels were observed. Consistently, expression levels of activated p53 (p-p53^Ser15^) as well as cell apoptosis marker cleaved caspase-3 and cleaved caspase-9 were also upregulated in the anti-DOG1 antibody treated HT-29 cells (**Figures 3H, I**).

In summary, our data suggest that anti-DOG1 antibody is able to induce apoptosis and inhibit cell migration and invasiveness in a p53-dependent apoptotic manner.

### Generation and characterization of the anti-DOG1 ADC

**Figure 4:**
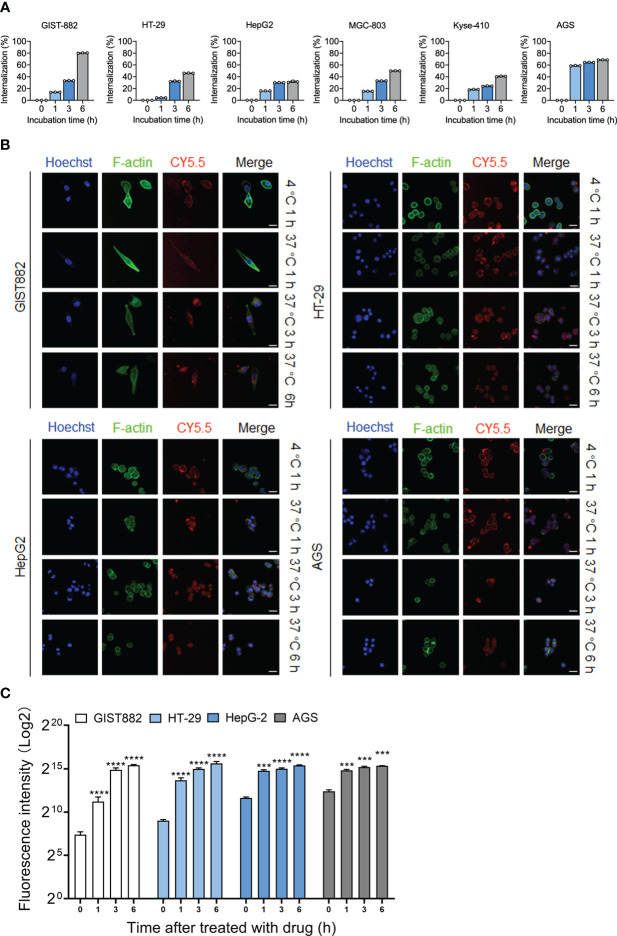
DOG1 could mediate the internalization of anti-DOG1 antibodies. **(A)** The internalization rate of the anti-DOG1 antibody was calculated using the formula [1-MFItime/MFIcontrol]×100%. DOG1 on the cell surface was detected by flow cytometry (n=3); **(B)** Immunofluorescence microscopy observation of the endocytosis of Cy5.5-labeled anti-DOG1 DM4 ADC mediated by DOG1 protein in GIST882, HT-29, HepG2, and AGS cell lines. The results are from 0 h, 1 h, 4 h and 8 h. Antibodies were stained with Cy5.5, rhodamine-labeled phalloidin was used to visualize the actin cytoskeleton (green), and Hoechst (blue) was used for nuclear staining. Scale bar, 25 µm; **(C)** Bar graph showing quantitative analysis of protein expressions internalization rate of the immunofluorescence images (n=3). ***, P <0.001; ****, P <0.0001.

Although the anti-DOG1 antibody demonstrated inhibitory effects on tumor cells, the consequences were not ideal. We aim to generate ADCs with anti-DOG1 to improve the therapeutic efficacy. To test this hypothesis, the internalization of the DOG1-antibody complex induced by the anti-DOG1 antibody was first determined. Specifically, cells were incubated with anti-DOG1 antibody for 1, 3, and 6 hours respectively followed by quantification of the DOG1 (+) cells by flow cytometry. Notably, treatment with the anti-DOG1 antibody could induce rapid DOG1-mediated internalization within 6 hours in human alimentary tract cancer cells, with the internalization percentage ranges from ~30% to ~80% (**Figure 4A**; **Figure S5**). The results further support the potentiality of anti-DOG1 as an effective “driver” of the ADC compounds.

To generate anti-DOG1 ADC, the highly potent microtubule inhibitor DM4 was conjugated to the anti-DOG1 antibody with SPDB as the linker (**Figure S6A**) (32). Specifically, the conventional lysine conjugation method was applied to conjugate SPDB-DM4 to lysine residues exposed at the surface of the anti-DOG1 antibody. The affinity of the anti-DOG1 ADC to the target was determined by surface plasmon resonance (SPR, BiaCore X100, GE) (**Figure S6B**). The drug-to-antibody ratio (DAR) value of the anti-DOG1-DM-ADC compound was 3.55 as measured by liquid chromatography–mass spectrometry, which was previously shown as an optimal functional attribute of ADCs (**Figure S6C**) (32).

Next, we applied confocal microscopy to visualize the dynamic internalization process of anti-DOG1-DM-ADC (33). The DOG1 high expressing cell lines (GIST882, HT-29, HepG2, and AGS) were initially incubated with anti-DOG1-DM-ADC at 4°C. The anti-DOG1 antibodies were stained with Cy5.5. The fluorescence signalings were then monitored dynamically under confocal microscopy. After incubation at 4°C for 1h, the conjugates virtually bound to cell membranes. After incubation at 37°C for 1h, amounts anti-DOG1-DM-ADC which bound to the cell membrane increased and endocytosis also occurred. After incubation at 37°C for 3h, the amounts of the complex binding to the surface of the cell membrane began to decrease, and more compounds appeared in the superficial cytoplasm. After incubation at 37°C for 6 h, the compounds were endocytosed into the deep cytoplasm, and few antibodies remained in the superficial distribution throughout the cell (**Figure 4B**). Quantification of the ADC internalization were characterized by using image J software to measure fluorescence intensity (**Figure 4C**).

In brief, the results demonstrate that the duration of DOG1-antibody complex binding to the cell surface suffices the internalization of payload drugs. The anti-DOG1-DM4-ADC can be rapidly endocytosed *via* DOG1 induced internalization.

### The anti-DOG1-DM4-ADC led to tumor inhibition *in vivo* and *in vitro*

**Figure 5:**
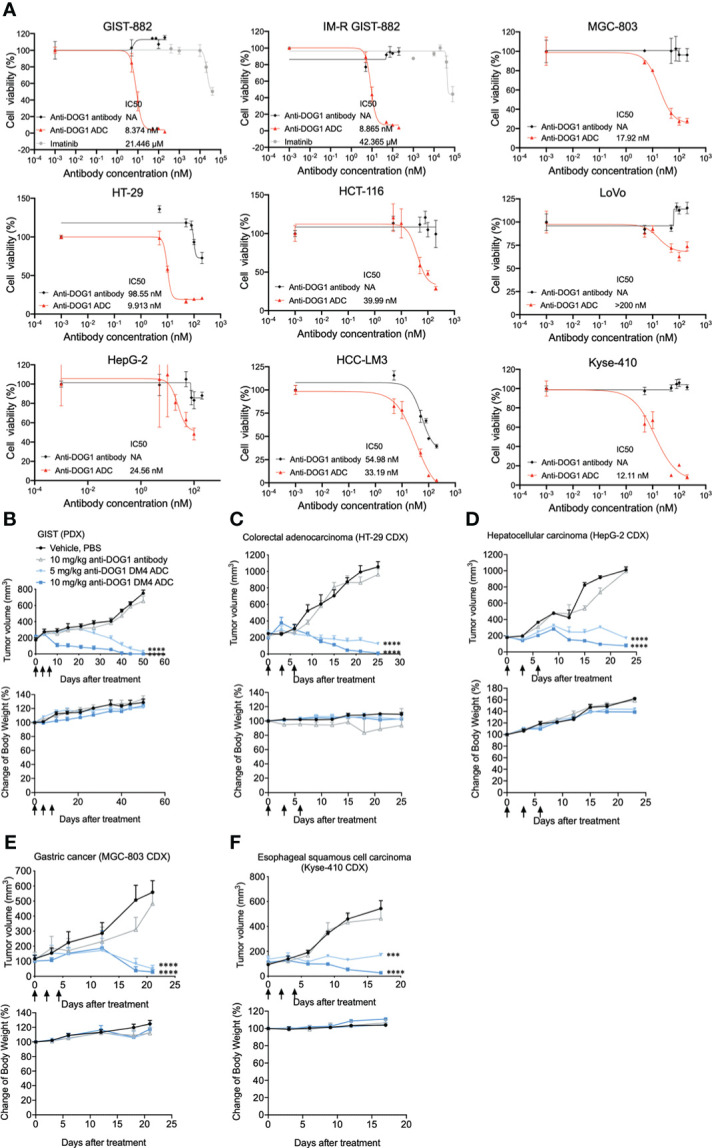
Anti-DOG1 ADCs showed potent *in vitro* and *in vivo* anti-tumor efficacy in multiple types of gastrointestinal tumor. **(A)** GIST882 and IM-resistant GIST882 cell lines were incubated with increasing concentrations of IM, unconjugated anti-DOG1 antibodies and anti-DOG1ADCs for 72 h. HT-29, HCT-116, LoVo, HepG2, HCC-LM3, MGC-803, and Kyse-410 cell lines were incubated with increasing concentrations of unconjugated anti-DOG1 antibodies and anti-DOG1 ADCs for 72 h. The cytotoxicity was calculated by IC_50_. NA: not active; **(B)** GIST PDX model (n=5); **(C)** HT-29 CDX model (n=5); **(D)** HepG2 CDX model (n=7); **(E)** MGC-803 CDX model (n=5); **(F)** Kyse-410 CDX model (n=5) were i.v. dosed Q3Dx3 as indicated (arrow) with vehicle, unconjugated anti-DOG1 antibodies at 10 mg/kg and anti-DOG1 ADCs at 5 or 10 mg/kg. Data from the tumor growth studies are depicted as the mean ± SEM. Compared with the control group by one-way ANOVA. ***P <0.001; ****P <0.0001. The body weight data are depicted as the mean ± SEM.

To explore the cytotoxic property of the anti-DOG1-DM4-ADC, the panel of human alimentary tract cancer (GIST, HCC, ESCA, GSAD and COAD) cell lines was first applied. Cancer cells were treated with anti-DOG1-DM4-ADC or naked anti-DOG1 antibody (as control) at different concentrations for 72 hours. Viable cell counts were determined with RTCA assays and the IC_50_ values were calculated. Overall, the anti-DOG1-DM4-ADC demonstrated significant cytotoxicity in a time- and dose- dependent manner with relatively low IC_50_ values (8nM ~ 40nM) among the various cell lines. In contrast, the unconjugated anti-DOG1 antibody was not active in most of the tested cell lines (**Figure 5A**; **Figure S7**). Additionally, as shown in **Figure 5A**, the anti-DOG1-DM4-ADC showed similar cytotoxic effects on both Imatinib sensitive GIST cells (GIST-882) and Imatinib resistant GIST cells (IM-R GIST-882). Of note, the cytotoxic efficacy was reduced in the LoVo human COAD cell line, which had low expression of membrane DOG1.

Next, we investigated the therapeutic efficacy of the anti-DOG1-DM4-ADC in murine xenograft models. The NOD/SCID/IL-2Rγ(null) mice (NSG mice were used to establish patient derived xenograft (PDX) models and the nude mice were used to establish cell line derived xenograft (CDX) models. After tumor inoculation, the tumor bearing mice were intravenously injected with naked anti-DOG1 antibody or a high/low dose of the anti-DOG1 ADC, and the vehicle control group was administered with PBS. Tumor volumes were used as the main indicator for the measurement of tumor growth. Compared with the vehicle and anti-DOG1 antibody treated group, the anit-DOG1-DM4-ADC showed a pronounced growth-inhibitory effect across GIST, COAD, HCC, STAD, and ESCA murine xenograft models (**Figures 5B-F**; **Table S1**).

Overall, our results indicate that anti-DOG1-DM4-ADC effectively inhibits tumor growth *in vitro* and *in vivo* among various alimentary tract cancer types.

### Anti-DOG1-DM4-ADC inhibits liver metastasis of colon cancer

**Figure 6:**
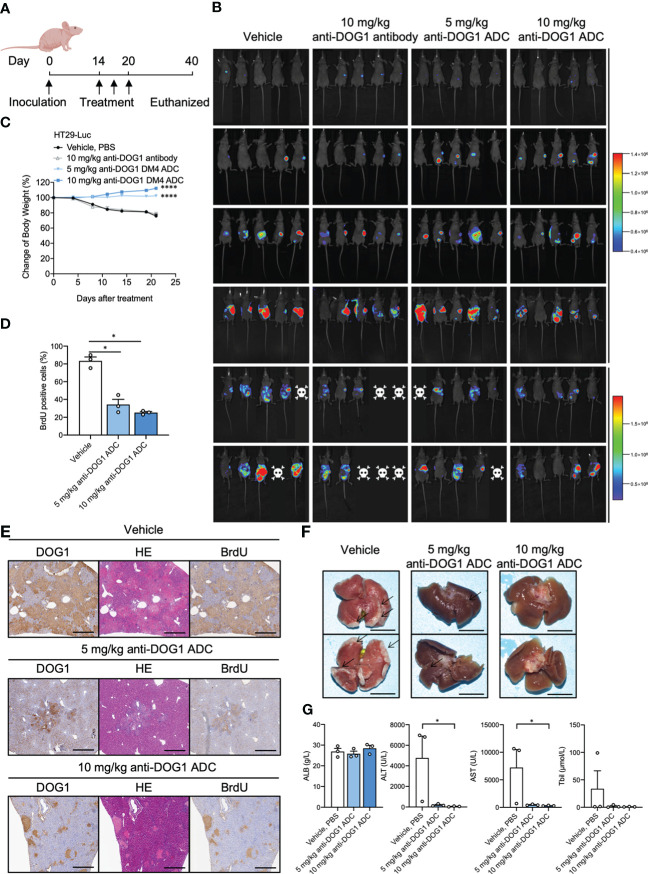
Anti-DOG1 antibody inhibited the experimental liver metastasis model of colon cancer. **(A)** Timeline of drug administration of the treated mice; **(B)** Bioluminescence on Day 1 to 35 post-HT-29-Luc cell injection; **(C)** The change in body weight during the experiment was calculated as the percent change in weight compared with the baseline measurement. Values are the mean ± SEM; n =5 mice per group; compared with the control group by one-way ANOVA; **(D)** BrdU positive rate of liver tissue on Day 35. Values are the mean ± SEM; n=3 mice per group; compared with the control group by one-way ANOVA; **(E)** DOG1, HE and BrdU IHC staining for liver tissue on Day 35; **(F)** Representative images of liver in the HT-29-Luc-bearing mice on Day 35; **(G)** The graph depicted ALB, ALT, AST Tbil on Day 40 after the inoculation. Values are the mean ± SEM; n=3 mice per group; compared with the control group by Friedman test or Kruskal-Wallis test. *P < 0.05; ****P <0.0001.

To investigate the anti-tumor effects of anti-DOG1 ADC on liver metastasis from colorectal cancer, we established a murine liver metastasis model. Specifically, luciferase expressing colon cancer cell lines HT-29 (HT-29-Luc) were injected into the spleen of *nude* mice. Mice were treated with unconjugated anti-DOG1 antibody or high/low concentration of anti-DOG1-DM4-ADCs 14, 17, and 20 days respectively after inoculation. The vehicle (PBS) was dosed as the control in parallel (**Figure 6A**). *In vivo* imaging system was applied to measure the tumor growth. To our excitement, anti-DOG1-DM4-ADC significantly inhibited the tumor cell metastasis and tumor growth as determined by bioluminescence imaging in the mouse livers. As expected, the results demonstrated that the liver metastasis were significantly decreased or not observable by bioluminescence imaging, accompanied by slight weight changes which generally means low systemic toxicity of the drug (**Figures 6B, C**). The proliferation rates of tumor cells were also inhibited according to BrdU IHC staining (**Figures 6D, E**). In addition, the DOG1 positive area percentage was significantly decreased in the anti-DOG1-DM4-ADC treated liver tissues (**Figure 6E**) compared to the vehicle treated liver tissues. Consistently, in the low concentration anti-DOG1-DM4-ADC treated group, only a few tumor nodules were observed macroscopically, while virtually no tumor nodule was visualized in the high concentration anti-DOG1-DM4-ADC treated group (**Figure 6F**). Of note, the anti-DOG1-DM4-ADC also demonstrated the capacity of preserving liver function, with signification reduction of serum levels of ALT, AST and total bilirubin (**Figure 6G**). Reference values for ALB, ALT, AST and total bilirubin from healthy animals are 27.7 ± 10.34g/L, 39.55 ± 6.49U/L, 114.45 ± 19.71 U/L, 1.8 ± 12.93μmol/L, respectively.

In summary, our results suggest that the anti-DOG1-DM4-ADC is effective for treating liver metastasis of colon cancer with protective effects on liver function.

## Discussion

Since Paul Ehrlich first proposed the concept of ADCs in the beginning of 20^th^ century, ADCs have been developed for decades, and become a class of precise targeting drugs with great potential against tumor. Until now there have been 14 ADC drugs approvals by FDA for both hematological malignancies and solid tumors worldwide (34). In addition, there are over 100 ADC candidates in the different stages of clinical trials at present. However, although variety of ADCs have been approved clinically or on clinical trials stage, the tumors they target are limited. Thus, there is a great need to identify novel targets to expand the application of ADCs for the treatment of primary tumors and metastasis.

To Develop ideal ADC drug, one must consider various key factor which includes selecting appropriate antigen that expressing on surface of tumor cell, navigating for ADCs to identify tumor cells. DOG1, one of the major components of the calcium-activated chloride channels expressed in the plasma membranes, is expressed in a wide variety of tumors surface but low or not in normal tissues, could be a potential therapeutic target. However, DOG1 has been used as a common diagnostic marker for gastrointestinal stromal tumors (GISTs), no evidence demonstrates that DOG1 could be therapeutic target. In this study, we confirmed that DOG1 was a surface marker highly expressed in gastrointestinal tumors including colon cancer, liver cancer, esophageal cancer and GIST. Anti-DOG1 mono antibody has shown a good internalization efficiency and certain therapeutic effect on tumors. The excellent internalization efficiency of the anti-DOG1 antibody and the feature of widely expression of DOG1 in many tumors, make DOG1 a great potential to be an ADC drug which, theoretically, could be a broad-spectrum anti-tumor drug against DOG1 positive cancer.

Although it is steadily declining in incidence, cancer of the alimentary system (esophageal, stomach, liver and colon) remains one of the most common and deadly neoplasms worldwide and is still a major challenge for cancer therapeutic options (35–38).. Despite various conventional therapeutic options, such as chemotherapy, radiotherapy, and surgical approaches, the survival rates remain notably low for patients with advanced disease (39). Further, due to various epidemiological backgrounds and genetic and epigenetic aberrations, clinical implementation of novel targeted drugs is limited. Lack of novel targeted drugs/therapeutic strategy is still one of the problems in the treatment of digestive system cancer. In addition, with advances in screening methods and treatment, the mortality rate of CRC in 2016 has declined by about half since the mid-1980s in the USA (40, 41). In addition to surgery and chemotherapy, targeted therapy (such as VEGF, EGFR, PD-1, CTLA-4. HER2, MEK, BRAF) has offered optional approaches to prolong overall survival for metastatic CRC patients (42). However, the inadequate response to therapy and poor prognosis correlates to CRC molecular heterogeneity. Resistance to targeted therapy could be acquired in patients through various mechanisms related to the target protein, such as gain-of-function mutations, activation of bypass signaling pathways, and crosstalk between associated pathways, resulting in poor efficacy and even disease progression (43–47). So especially for patients with metastatic lesions, more effective approaches for medical intervention are required. On the other hand, CTCs are now considered to be a very important risk factor for tumor recurrence and metastasis, completely elimination of CTCs is an important indicator for evaluating anti-tumor efficiency of new developing therapeutic drugs recently, but there are few strategies directly targeting CTCs. Due to highly expression of DOG1 in CTCs derived from colon cancer patients, we propose a hypothesis that our novel anti-DOG1-DM4-ADC may efficiently directly target these DOG1 positive CTCs, therefor, prevent tumor recurrence or metastasis. In conclusion, our study demonstrates that the anti-DOG1-DM4-ADC construct is effective for treating alimentary tract cancers, providing alternative therapeutic approaches for selected metastatic colon cancer patients.

The distinguishable expression of targets in tumor and normal tissue is essential for the manageable safety profiles of ADCs (48). In this report, IHC screening of the pan-gastrointestinal cancer TMAs showed that DOG1 is highly expressed in GIST and esophageal squamous cell carcinoma, which is consistent with previously published research (1, 49). Our research broadens the DOG1 expression profiles in colon cancer, liver cancer, gastric cancer, colon adenocarcinoma, gastric adenocarcinoma, cardia adenocarcinoma, and liver metastatic cancer. DOG1 is expressed at low levels or even not expressed in corresponding normal tissues, which is essential for the on-target toxicity of ADCs. All these IHC profiles supported that DOG1 could be developed as a target for ADCs. This development would broaden the activity against cancers overexpressing DOG1, where the target may not be a driver because ADC activity is driven primarily by the DM4 cytotoxin (50).

The key characteristic of the target antigen for ADC drugs is to bind the antibodies on the surface of tumor cells to enrich cytotoxic drugs in tumor cells (48, 51). On this basis, we used qRT-PCR and flow cytometry to screen corresponding cell surface DOG1^+^ tumor cell lines. Another key characteristic of the target antigen is to mediate the endocytosis of the antigen-ADC complex by tumor cells (48). We observed the endocytosis of fluorescence-labeled anti-DOG1 ADCs in GIST882, HT-29, HepG2, and AGS cell lines with a confocal microscope. In addition, the unconjugated anti-DOG1 antibody could decrease proliferation, and migration in cancer cells, generating combined activity after conjugated with the cytotoxic drug.

In summary, we successfully constructed a new type of ADC drug, anti-DOG1 ADC. *In vivo* and *in vitro* efficacy studies showed that the conjugate can correctly identify and efficiently kill tumor cells highly expressing DOG1. In addition, treatment with anti-DOG1 ADCs in liver metastasis models suggested potential liver function protective effects. The above results suggest that anti-DOG1 ADCs may be promising first-in-class therapeutic molecules for DOG1-positive tumors such as GIST, colon cancer, liver cancer, gastric cancer and esophageal cancer and may be used in preventive treatment protocols for inhibiting recurrence after curative resection of liver metastases of colorectal origin.

## Conclusion

In summary, anti-DOG1-ADC exhibits potent and dose-dependent anti-tumor activity in xenograft models compared with naked antibody in a DOG1-dependent manner with acceptable toxicity. Altogether, our findings emphasize the potential efficacy of anti-DOG1-ADC as a first-in-class treatment option for patients with DOG1-expressing alimentary tract tumors and liver metastasis.

## Data availability statement

The original contributions presented in the study are included in the article/**Supplementary Material**. Further inquiries can be directed to the corresponding author/s.

## Ethics statement

The animal study was reviewed and approved by Animal Ethical and Welfare of West China Hospital, Sichuan University.

## Author contributions

WL and JY conceived and supervised this research. YW designed the research plan and performed most of the experiments and/or data analyses. YW and HW contributed to the antibody design and construction. WTL and SS synthesized the bullet drug and linker. XC, XD and LL contributed to the immunohistochemistry and data assays. YX and MW contributed to the RT-PCR trial. XD and TY contributed to molecular biology experiments. YW, WTL, HW, and XC conducted animal *in vivo* experiments. WTL and YW collected the data and generated the figures. YW and HW wrote the first draft of the manuscript. YL contributed to writing-review and editing. All authors contributed to the article and approved the submitted version.
