# Supplementary material for: DOG1 as a novel antibody-drug conjugate target for the treatment of multiple gastrointestinal tumors and liver metastasis
Source: Front Immunol. 2023 Jan 26;14:1051506. doi: 10.3389/fimmu.2023.1051506 (PMC9909470; doi:10.3389/fimmu.2023.1051506)
Supplement: Supplementary file 1 [file DataSheet_1.docx]

**DOG1 as a novel antibody-drug conjugate target in for the treatment of multiple gastrointestinal tumors and liver metastasis**

**Running Title:** DOG1 as an ADC target for GI tumor therapy

Yangping Wu^1,2^, Wenting Li^2^, Xiangzheng Chen^3,2^, Haichuan Wang^3^, Siyuan Su^5^, Ying Xu^1^, Xiangbing Deng^4^, Tinghan Yang^4^, Mingtian Wei^4^, Li Li^5^, Jinliang Yang^2^,* and Weimin Li^1^,*

^1^ Department of Respiratory and Critical Care Medicine, West China Hospital, Sichuan University, Chengdu, China

^2^ State Key Laboratory of Biotherapy and Collaborative Innovation Center for Biotherapy, West China Hospital, Sichuan University, Chengdu, China

^3^ Department of Liver Surgery & Liver Transplantation, West China Hospital, Sichuan University, Chengdu, China

^4^ Department of Gastrointestinal Surgery, West China Hospital, Sichuan University, Chengdu, Sichuan, China;

^5^ Department of Chemistry, University of Illinois at Chicago, Chicago, United States

^6^ Department of Pathology, West China Hospital, Sichuan University, Chengdu, China

**Corresponding Author**: Weimin Li, Department of Respiratory and Critical Care Medicine, West China Hospital, Sichuan University, 37 Guoxue Xiang, Chengdu, Sichuan, China, 610041. Phone: +86 28 85423998, Fax: +86 28 85582944, Email: weimin003@163.com; Or Jinliang Yang, State Key Laboratory of Biotherapy and Cancer Center/Collaborative Innovation Center for Biotherapy, West China Hospital, Sichuan University, 3-17 People Road, Chengdu, Sichuan, China, 610041. Phone: +86 28 85502796, Fax: +86-28-85502796, Email: [jinliangyang@scu.edu.cn](mailto:jinliangyang@scu.edu.cn)

**Supplementary figures**


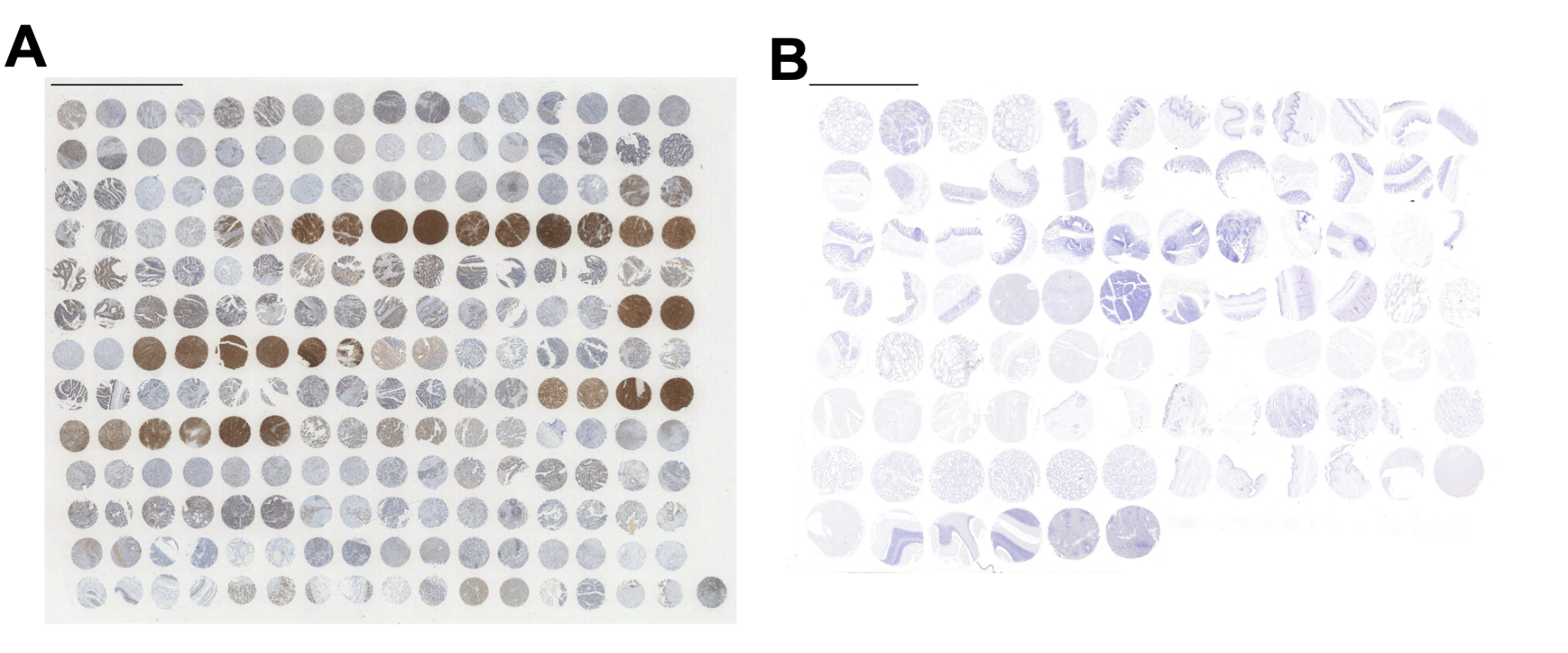


**Figure S1. DOG1 is expressed in gastrointestinal cancer but not or low in normal tissues.** **A,** DOG1 expression in gastrointestinal cancer, including GIST, and esophageal, colon, liver and gastric cancer. **B,** DOG1 expression across 27 distinct normal tissue. Scale bars equal 5 mm.

**
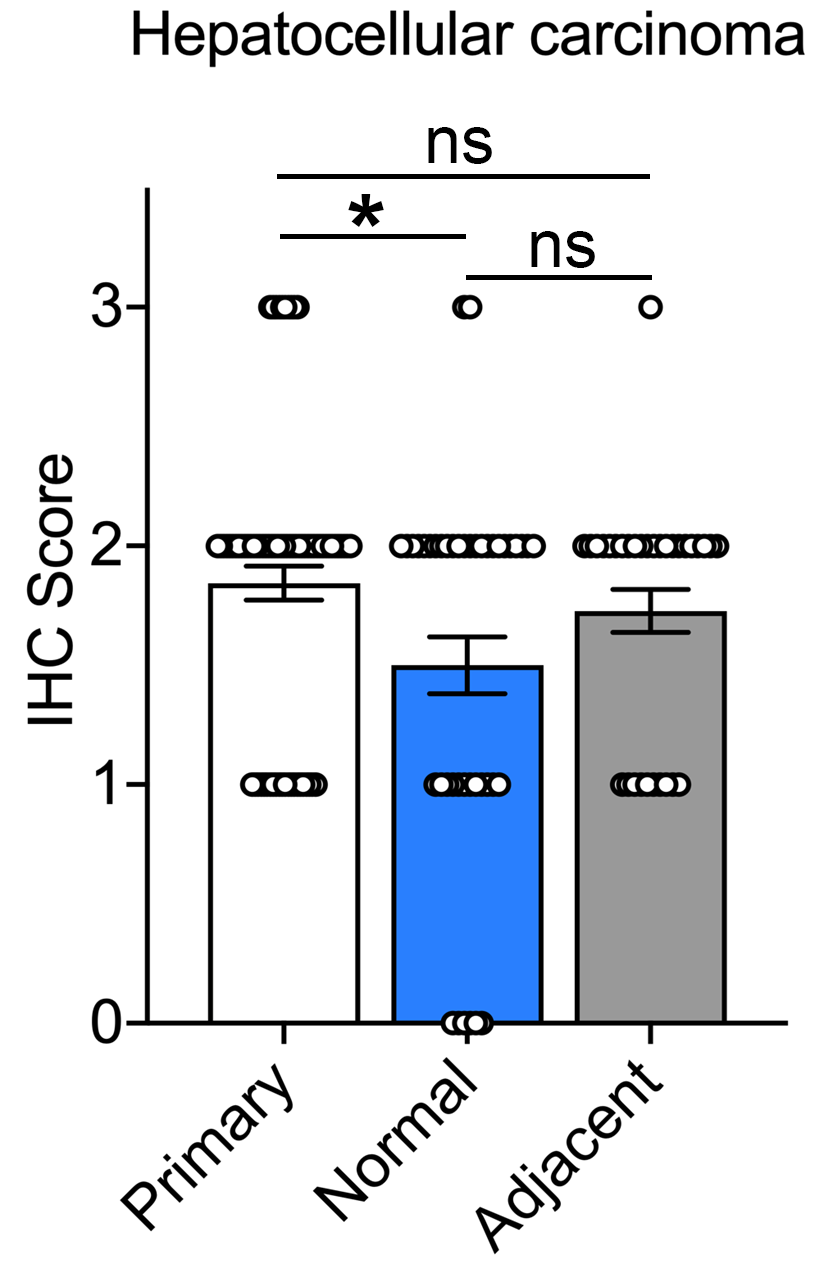
**

**Figure S2. IHC scores of liver cancer TMAs.** Primary: primary tumor (n=77); Normal: healthy normal tissue (n=44); Adjacent: normal tissue adjacent to the tumor (n=33). Kruskal-Wallisis test. Comparison within groups: *P < 0.05.


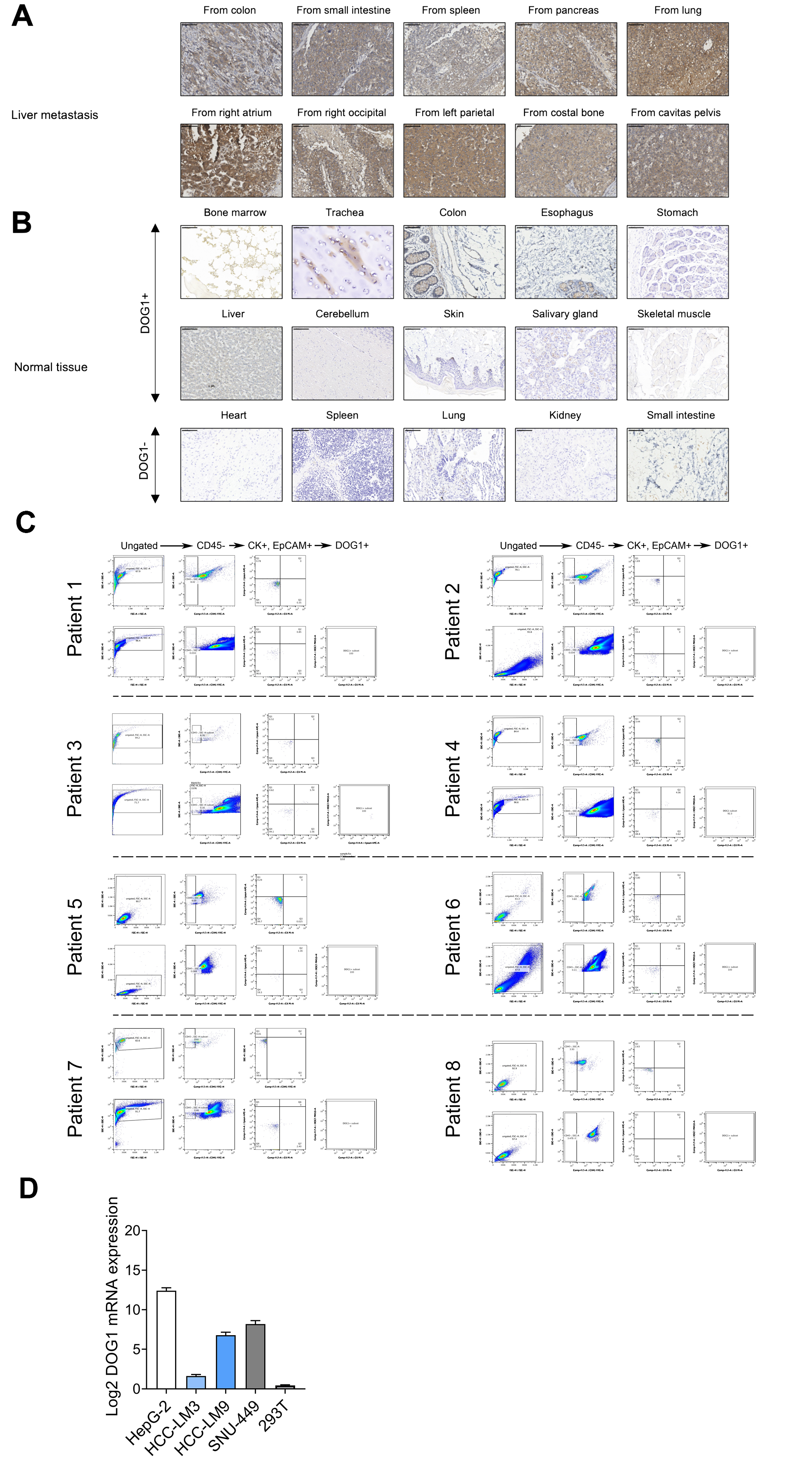


**Figure S3. The expression of DOG1 in tumor tissues of clinical patients and cell lines. A:** Representative immunohistochemical images for DOG1 protein expression in various neoplasms of alimentary tract; **B:** healthy normal tissue specimens. Positive IHC staining for DOG1 is indicated by a brown precipitate. Scale bar, 50 µm; C: CTC from plasm samples of 8 colon cancer patient detected by FACS; D: mRNA expression level of different HCC cell lines detected by qPCR, 293T was negative control (DOG1-).

**
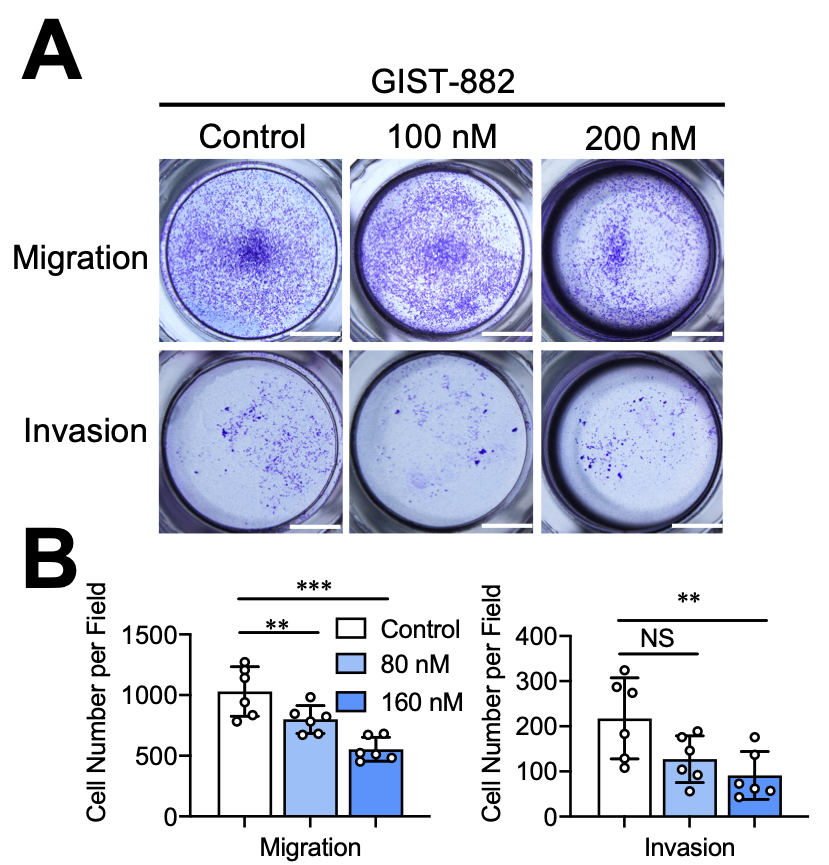
**

**Figure S4. Anti-DOG1 antibody inhibited cell migration and invasion in GIST-882 cells**. **A**: Migration (without Matrigel) and invasion (with Matrigel) of HT-29 cells were suppressed by the anti-DOG1 antibody compared with the control as shown by Transwell assays. Representative images are shown. Scale bar, 100 µm; **B:** Bar graphs of panel **A** are shown. Kruskal-Wallisis test, comparison within groups: **P < 0.01, ***P＜0.001.

**
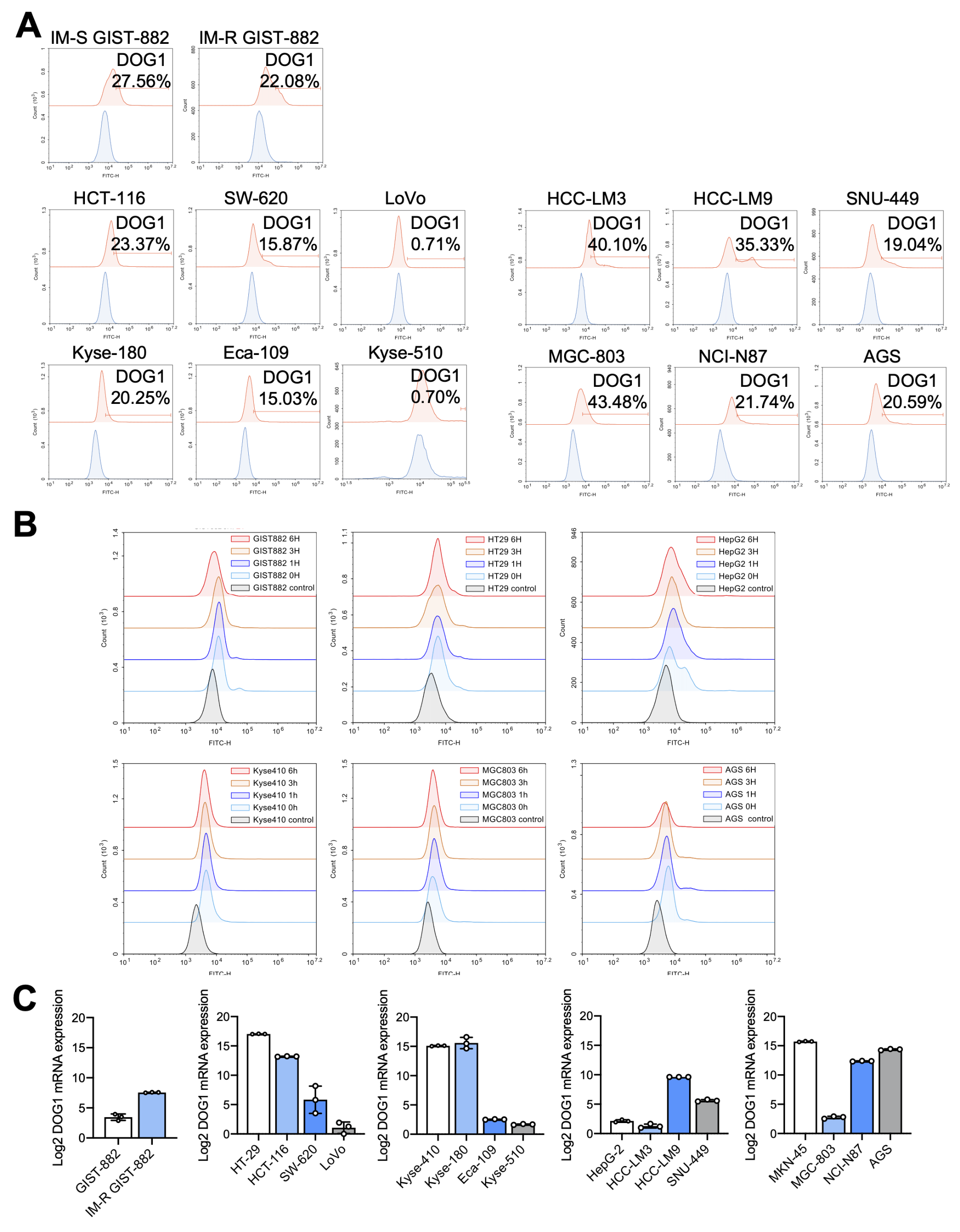
 Figure S5. DOG1 surface expression in internalization rate assays by flow cytometry**.

**
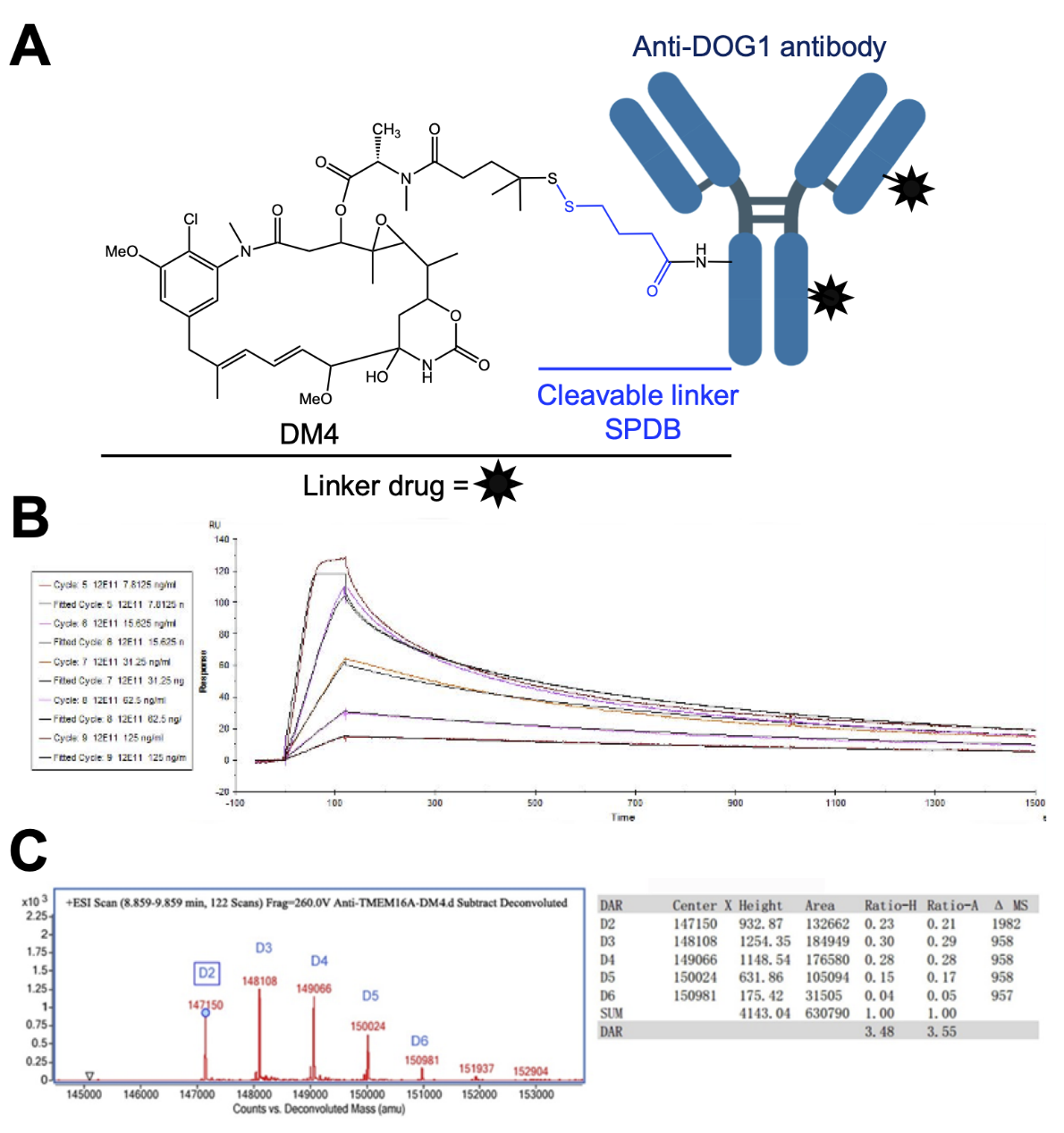
**

**Figure S6. Characterization of anti-DOG1 DM4 ADC and anti-DOG1 antibody**. **A**: Schematic of anti-DOG1 DM4 ADC; **B:** The real-time binding profile between anti-DOG1 antibody and DOG1 peptide fragment characterized by SPR Biacore. The ka of anti-DOG1 antibody is 7.266×10^9^ M^-1^s^-1^, the kd is 22.44 s^-1^, and the K_D_ is 3.088×10^-9^ M; **C:** The DAR (Drug-Antibody Ratio) was determined by LC-MS. Average DAR=3.55.

**
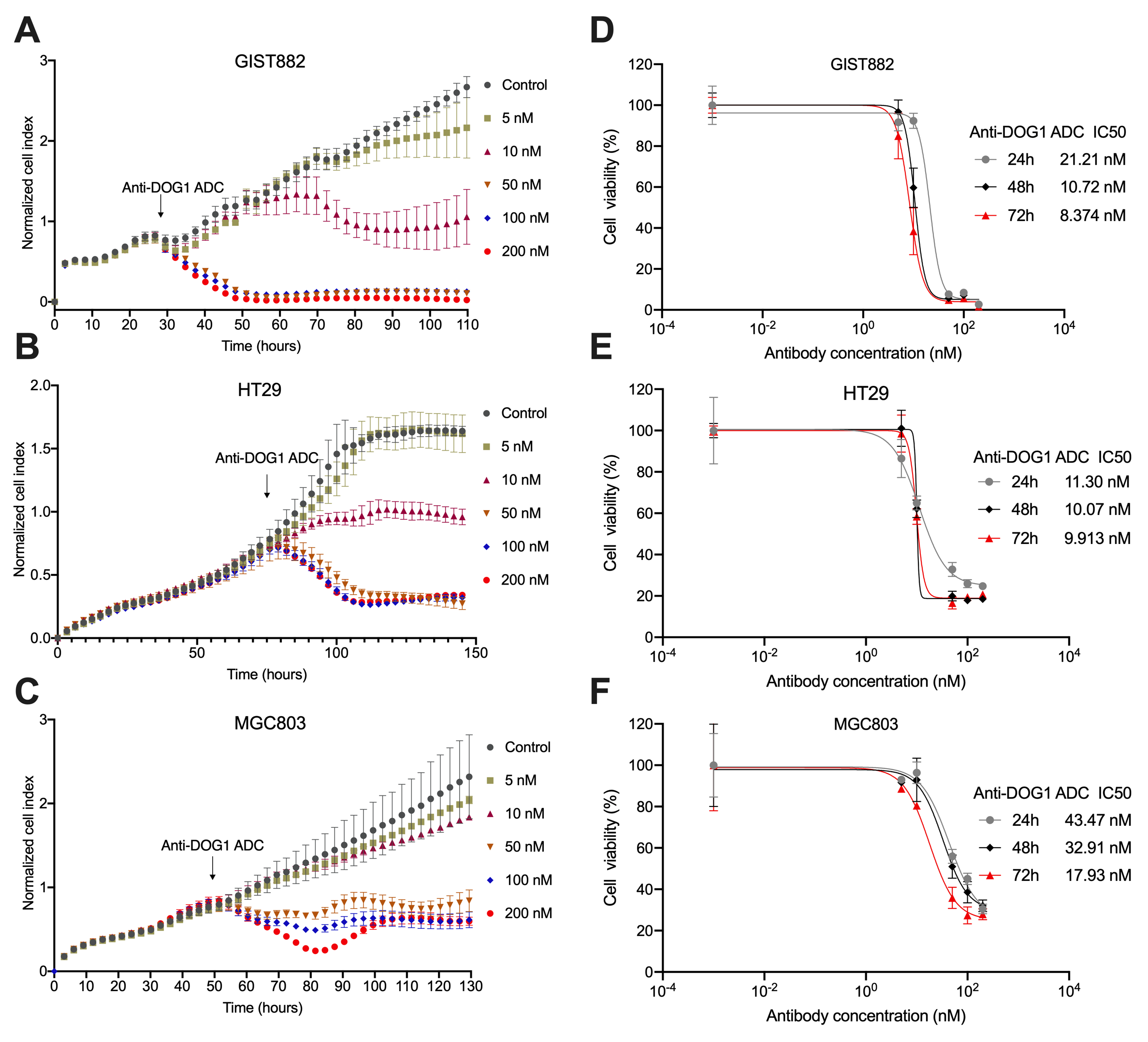
**

**Figure S7. Anti-DOG1 DM4 ADCs showed time-dependent cytotoxicity. A-C,** Cell Index represents the change in electrical impedance over time. Each cell line was plated in triplicate and error bars indicate standard deviation of those three wells. The graphs shown are representative of 3 independent runs. **D-F,** In vitro cytotoxicity of anti-DOG1 DM4 ADCs upon incubation with GIST882, HT29 and MGC803 for 24 h, 48 h and 72 h.

**Supplementary Tables**

**Table S1.**

In Vivo anti-tumor efficacy of the anti-DOG1 ADCs in a panel of murine xenograft models representing multiple types of gastrointestinal tumor. %TGI, percent tumor growth inhibition; CR: complete response; PR: partial response

| **No.** | **Model** | **Cancer type** | **Cell surface DOG1+ (%)** | **%TGI** | | **CR rate (%)** | | **PR rate (%)** | |
| --- | --- | --- | --- | --- | --- | --- | --- | --- | --- |
|  |  |  |  | **5 mg/kg** | **10 mg/kg** | **5 mg/kg** | **10 mg/kg** | **5 mg/kg** | **10 mg/kg** |
| 1 | GIST PDX | GIST | 22.08 | 96.13 | 100 | 60 (3/5) | 100 (5/5) | 40 (2/5) | 0(0/5) |
| 2 | HT2-9 | Colon cancer | 32.75 | 76.95 | 99.61 | 0 (0/5) | 60 (3/5) | 0 (0/5) | 0 (0/5) |
| 3 | HepG2 | Hepatocellular carcinoma | 87.03 | 57.55 | 76.41 | 0 (0/7) | 0 (0/7) | 0 (0/7) | 0 (0/7) |
| 4 | MGC-803 | Gastric cancer | 43.49 | 100 | 93.48 | 20 (1/5) | 0 (0/5) | 0 (0/5) | 0 (0/5) |
| 5 | Kyse-410 | Esophageal cancer | 20.94 | 85.17 | 85.97 | 0 (0/5) | 0 (0/5) | 40 (2/5) | 0 (0/5) |

**Supplemental experimental procedures**

**Preparation and characterization of anti-DOG1 antibodies**

Human DOG1 ECD2 (amino acids 541 to 556) was produced by ChinaPeptides. Five Balb/c mice (HFK Biotechnology) were injected intramuscularly with human DOG1 ECD2 in Quick-Antibody-Mouse5W adjuvant (KX0210041, Biodragon). Serum titers were evaluated by standard enzyme-linked immunosorbent assays (ELISAs) after two injections on day 35. Splenic B cells harvested from a total of five mice were fused with mouse myeloma cells (P3X63.Ag8.653, American Type Culture Collection) by PEG1500 (10783641001, Sigma-Aldrich). After 10 to 14 days, hybridoma supernatants were screened for antibody secretion by ELISAs. All positive clones were then expanded and rescreened for human DOG1 ECD2 binding by ELISAs and FACS analysis. Hybridoma clone 3D7 was humanized to generate 3D7v2. Anti-DOG1 antibodies used in this article referred to 3D7v2. These antibodies were then generated in the FreeStyle 293-F mammalian cell transient expression system. Seven days after transfection, the expressed supernatant was collected, and protein purification was performed using a HiScreen MabSelect PrismA column (GE Healthcare) with an NGC system (Bio-Rad). The binding and dissociation constant of anti-DOG1 antibodies and affinity of human DOG1 ECD2 were determined by Biacore X100 (GE Healthcare) and analyzed with a 1:1 model.

**Antibody for immunoblotting**

Anti-DOG1 antibody was used at 0.5 µg/ml, anti-β-actin antibody (sc-69879, Santa Cruz Biotechnology) at 1:1000, anti-p53 antibody (2527T, Cell Signaling Technology) at 1:1000, anti-Phospho-p53 (Ser15) antibody (9286, Cell Signaling Technology) at 1:1000, anti- Caspase-3 antibody (9665, Cell Signaling Technology) at 1:1000, anti-Cleaved Caspase-3 antibody (9664, Cell Signaling Technology) at 1:1000, anti-Caspase-9 antibody (9508, Cell Signaling Technology) at 1:1000, anti-Cleaved Caspase-9 antibody (7237, Cell Signaling Technology) at 1:1000, anti-Notch2 antibody (A0560, ABclonal) at 1:2000, anti-Notch3 antibody (A13522, ABclonal) at 1:2000, anti-Notch4 antibody (A8303, ABclonal) at 1:2000, anti-Hes1 antibody (ab108937, Abcam) at 1:2000, anti-c-Myc antibody (A19032, ABclonal) at 1:2000, anti-Akt (pan) antibody (4691P, Cell Signaling Technology) at 1:1000, anti-mTOR antibody (2983P, Cell Signaling Technology) at 1:1000, IRDye 800CW Goat anti-Mouse IgG Secondary Antibody (926-32210, LI-COR) at 1:10,000, IRDye 680RD Goat anti-Rabbit IgG Secondary Antibody (926-68071, LI-COR) at 1:10,000.

**Immunohistochemistry (IHC) analysis**

IHC analysis of the tissue specimens and TMAs was performed by the anti-DOG1 antibody sp31 (ab64085, Abcam) and Anti-BrdU antibody (ab6326, Abcam). In brief, formalin-fixed, paraffin-embedded tissue and TMA specimens were deparaffinized, and antigen unmasking was performed in a PT Module (Thermo Scientific) with EDTA retrieval buffers (AR0023, Boster) at 99 °C for 15 min. Endogenous peroxidase was inhibited by treatment with 3% H_2_O_2_ in phosphate-buffered saline (PBS) for 4 min, and endogenous biotin was blocked with an Endogenous Biotin-Blocking Kit (E21390, Invitrogen). Endogenous immunoglobulin Gs (IgGs) were blocked with 5% bovine serum albumin (BSA)/PBS, and primary antibody was incubated for 60 min at 37 ℃. Biotinylated secondary antibody was incubated for 30 min at 37 ℃, followed by treatment with streptavidin-biotin complex (SABC) (SA1022, Boster) for 30 min at 37 ℃. Antibody binding was detected with metal-enhanced 3,3'-diaminobenzidine (DAB) (AR1022, Boster) for 5 min at room temperature, and sections were counterstained with Mayer’s hematoxylin (AR0005, Boster). Cell membrane staining was used to assess positivity for DOG1. Slides were examined independently by two blinded pathologists who assigned an IHC score of 0 (no staining in >90% of tumor cells), 1 (positive staining in <10% of tumor cells), 2 (positive staining in >10% and ≤50% of tumor cells), or 3 (positive staining in >50% of tumor cells). IHC scores ≥2 were considered positive.

**Real-Time Quantitative RT-PCR**

Primers were synthesized by Sangon Biotech. Total RNA was extracted from diﬀerently treated cells using RNA-easyTM Isolation Reagent (R701, Vazyme). PCR reactions were performed by Platinum SYBR Green qPCR SuperMix-UDG with ROX (11744500, Invitrogen) and run on QuantStudio 3 Real-Time PCR Systems (Thermo Fisher). The conditions for the amplification reaction were as follows: 50 ℃ for 2 min, 95 ℃ for 2 min, followed by 40 cycles of 95 ℃ at 15 s and 60 ℃ for 30 s. The melt curve protocol was 5 seconds each at 0.5 ℃ increments from 65 ℃ to 95 ℃. Each sample was processed in triplicate. Primer sequences that were designed on the basis of the published human gene sequences. The relative mRNA expression level was calculated by the 2^-ΔΔCt^ method and normalized to the 18S or GAPDH housekeeping gene.

**PCR Primer sequences.**

DOG1 (sense: 5'-GCTGACATGGAGAGATCGGYYC-3', antisense: 5'-TGGAGATTCTGTAGATGATGACGC-3')

18S (sense: 5'- TAGAGGGACAAGTGGCGTTC-3', antisense: 5'-CGCTGAGCCAGTCAGTGT-3').

P53 (sense: 5'-CAGCACATGACGGAGGTTGT-3', antisense: 5'- TCATCCAAATACTCCACACGC-3')

Notch1 (sense: 5'-GAGGCGTGGCAGACTATGC-3', antisense: 5'-CTTGTACTCCGTCAGCGTGA-3')

Notch2 (sense: 5'-CAACCGCAATGGAGGCTATG-3', antisense: 5'-GCGAAGGCACAATCATCAATGTT-3')

Notch3 (sense: 5'-TGGCGACCTCACTTACGACT-3', antisense: 5'-CACTGGCAGTTATAGGTGTTGAC-3')

Notch4 (sense: 5'- TGTGAACGTGATGTCAACGAG-3', antisense: 5'-ACAGTCTGGGCCTATGAAACC-3')

RBP-Jκ (sense: 5'-CGGCCTCCACCTAAACGAC-3', antisense: 5'-TCCATCCACTGCCCATAAGAT-3')

Hes1 (sense: 5'-TCAACACGACACCGGATAAAC-3', antisense: 5'-GCCGCGAGCTATCTTTCTTCA-3')

TGF-β1 (sense: 5'-GGCCAGATCCTGTCCAAGC-3', antisense: 5'-GTGGGTTTCCACCATTAGCAC-3')

TGF-β2 (sense: 5'-CAGCACACTCGATATGGACCA-3', antisense: 5'-CCTCGGGCTCAGGATAGTCT-3')

SMAD2 (sense: 5'-CGTCCATCTTGCCATTCACG-3', antisense: 5'-CTCAAGCTCATCTAATCGTCCTG-3')

SMAD3 (sense: 5'-TGGACGCAGGTTCTCCAAAC-3', antisense: 5'-CCGGCTCGCAGTAGGTAAC-3')

SMAD4 (sense: 5'-CTCATGTGATCTATGCCCGTC-3', antisense: 5'-AGGTGATACAACTCGTTCGTAGT-3')

GAPDH (sense: 5'- GGAGCGAGATCCCTCCAAAAT-3', antisense: 5'- GGAGCGAGATCCCTCCAAAAT-3')

**Cell migration and invasion assays**

In vitro cancer cell migration and invasion activities were evaluated in Transwells. HT-29 and GIST-882 cells were trypsinized, washed and counted. For cell migration, 1×10^5^ cells in 200 µl of medium without FBS were seeded in the upper Transwell insert chamber containing a polycarbonate filter (3422, Transwell, Corning). DMEM (600 µl) with 10% FBS (Gibco) was added to the lower chamber, and the plates were incubated for 24 h at 37 °C in 5% CO_2_. For cell invasion, the Transwells were coated with Matrigel. The cells that did not migrate were removed from the top of the Transwell filters by scraping. The penetrated cells were fixed with paraformaldehyde, stained with crystal violet and counted under a stereomicroscope (SMZ18, Nikon). The cell number represents the migratory activity.

**Wound healing assays**

Cells were seeded in a 6-well plate until confluent, scraped with a sterile, 200μl tip and washed twice with fresh serum free media. After incubation with DMEM medium containing 1% fetal bovine serum the cells were photographed at 0 h, 24 h, and 48 h under an inverted microscope (TE2000, Nikon). The images were analyzed by the NIC Elements imaging software (Nikon).
